# Analysis of preoperative nutrition, immunity and inflammation correlation index on the prognosis of upper tract urothelial carcinoma surgical patients: a retrospective single center study

**DOI:** 10.1186/s12893-024-02496-y

**Published:** 2024-07-16

**Authors:** Yong Ou, Yang Zheng, Dong Wang, Shangqing Ren, Yisha Liu

**Affiliations:** 1 Department of Robotic Minimally Invasive Surgery Center, Sichuan Academy of Medical Sciences and Sichuan Provincial People’s Hospital and Affiliated Hospital of the University of Electronic Science and Technology of China, Chengdu, 610072 China; 2 Department of Urology, Xichang People’s Hospital, Xichang, Sichuan China; 3 Department of Pathology, Sichuan Academy of Medical Sciences and Sichuan Provincial People’s Hospital, Chengdu, China

**Keywords:** Upper tract urothelial carcinoma, Systemic immune-inflammation index, Prognostic nutritional index, Systemic inflammation response index, Albumin-to-alkaline phosphatase ratio, Lung immune prognostic index

## Abstract

**Background:**

SII, PNI, SIRI, AAPR, and LIPI are prognostic scores based on inflammation, nutrition, and immunity. The purpose of this study was to examine the prognostic value of the SII, PNI, SIRI, AAPR, and LIPI in patients with UTUC who underwent radical nephroureterectomy with bladder cuff excision.

**Materials and methods:**

Data of UTUC patients in Sichuan Provincial People's Hospital from January 2017 to December 2021 were collected. The optimal critical values of SII, PNI, SIRI, and AAPR were determined by ROC curve, and LIPI was stratified according to the dNLR and LDH. The Kaplan–Meier method was used to draw the survival curve, and Cox proportional hazard model was used to analyze the factors affecting the prognosis of UTUC patients.

**Results:**

A total of 81 patients with UTUC were included in this study. The optimal truncation value of PNI, SII, SIRI and AAPR were determined to be 48.15, 596.4, 1.45 and 0.50, respectively. Univariate Cox proportional hazard regression showed that low PNI, high SII, high SIRI, low AAPR and poor LIPI group were effective predictors of postoperative prognosis of UTUC patients. Multivariate Cox proportional hazard regression showed that high SII was an independent risk factor for postoperative prognosis of UTUC patients. According to ROC curve, the prediction efficiency of fitting indexes of PNI, SII, SIRI, AAPR and LIPI is better than that of using them alone.

**Conclusions:**

The SII, PNI, SIRI, AAPR, and LIPI was a potential prognostic predictor in UTUC patients who underwent radical nephroureterectomy with bladder cuff excision.

## Introduction

According to the tumor statistics released by the United States in 2019, urothelial carcinoma (UC) is the fourth leading malignant solid tumor in the world and the most common malignant tumor of the urinary system [1]. UC can be located in the lower urinary tract (bladder and urethra) or the upper urinary tract (renal pelvis and ureter). Upper tract urothelial carcinoma of the bladder (UCB) is the most common, accounting for 90–95%, while upper urinary tract urothelial carcinoma (UTUC) only accounts for 5–10% [2]. UTUC, including renal pelvis cancer and ureteral cancer, is characterized by occult onset, high invasiveness, high recurrence rate, and poor prognosi [3].

At present, the prognostic evaluation of UTUC mainly depends on TNM staging, tumor invasion and other common indicators. However, recent studies have shown that inflammation is closely related to tumors [4]. Inflammation provides a tumor microenvironment (TME) for tumors and promotes angiogenesis and cell proliferation through different mechanisms, playing an important role in the occurrence and development of tumors, as well as their late invasion and metastasis, thereby affecting the prognosis of patients. In recent years, the Systemic Immuno-Inflammation Index (SII) [5], based on lymphocyte, platelet, and neutrophil counts, has been developed and proved to be an accurate and powerful predictor of the survival and recurrence of tumors, such as hepatocellular carcinoma and prostate cancer [6, 7]. In addition, the Systemic Inflammation Response Index (SIRI) is a novel inflammation-related index based on three common inflammation-related indicators, including peripheral neutrophil, monocyte, and lymphocyte counts, and was first developed by Qi in 2016 [8]. This study reported SIRI to be a simple and practical inflammatory index for predicting the prognosis of patients with advanced pancreatic cancer. It has been reported in many studies that SIRI is associated with clinical and pathological features and prognosis of various tumors [9, 10].

Preoperative nutritional and immune status is associated with postoperative complications and the long-term prognosis of patients with malignant tumors [11]. In recent years, some studies have reported that new indicators based on inflammation and nutritional variables have been developed to predict the prognosis of patients with malignant tumors. The Prognostic Nutrition Index (PNI) is an early evaluation index targeting the perioperative surgical risk and nutritional status of patients undergoing gastrointestinal surgery [12]. PNI can be determined from total lymphocyte count and serum albumin level, which has been proven to be an effective prognostic marker for various malignant tumors [13, 14].

Albumin is a serum protein and can reflect the body’s nutritional status and inflammatory level of patients. Some studies have shown that hypoproteinemia is often closely related to the body's low immune ability [15]. Alkaline phosphatase is a common hydrolase which can be synthesized by tumor cells. It affects the inflammatory level of the body by adjusting the purine signal pathway and induces inhibitory immune response in the development of tumor [16]. Albumin-to-alkaline phosphatase ratio (AAPR) is the ratio of serum albumin to alkaline phosphatase, and its predictive value for patients' prognosis has been confirmed in many malignant tumors, including non-small cell lung cancer, renal cancer, and nasopharyngeal carcinoma [17–19].

Lymphocytes, neutrophils, and platelets play an important role in the immune process and inflammatory response of the tumor. For example, a variety of inflammatory factors released by neutrophils can stimulate the TME and promote the metastasis of tumor cells. The derived neutrophil to lymphocyte ratio (dNLR) can be considered an indicator of prognosis in patients with various tumors [20]. The concept of the lung immune prognostic index (LIPI) score was first proposed by Mezquita [21]. LIPI scores derived from dNLR and lactate dehydrogenase (LDH) can reflect the inflammatory state of the body. LIPI formed by the combination of dNLR and LDH can effectively inform the prognosis of tumors [22].

In this study, preoperative blood indicators of patients with UTUC were analyzed to calculate the relevant SII, SIRI, PNI, AAPR, and LIPI scores and assess their impact on the prognosis of patients with UTUC.

## Materials and methods

### Patient data

A total of 81 patients who underwent robot-assisted laparoscopic radical nephroureterectomy + cystsleeve (RNU) resection in the Sichuan Provincial People's Hospital from January 2017 to December 2021 and were confirmed as UTUC by postoperative pathology were selected retrospectively, including 48 cases of renal pelvic carcinoma and 33 cases of ureteral carcinoma (The date of retrospective access to data is from January 2017 to April 2023). The exclusion criteria were inflammatory diseases, liver diseases, autoimmune diseases, hematological diseases, other types of tumors, cardiovascular and cerebrovascular diseases, and patients lost to follow-up.

### Clinical and pathological data

Clinical data were age, sex, body mass index (BMI), and preoperative blood sample data. Pathological data included tumor stage, grade, and type. The 2017 TNM staging of UTUC was used for tumor staging, and tumor grading was based on the 2014 WHO classification. In addition, tumor types can be divided into papillary and invasive tumors according to tumor morphology under a tumor microscope and whether it infiltrates epithelial tissue and subtypes. Pathological specimens were interpreted by experienced pathologists at Sichuan Provincial People's Hospital [10, 23–26].

$$\text{PNI }=\text{ Albumin concentration }(\mathrm{g}/\mathrm{L}) + 5\times \text{lymphocyte count }(\times 109/\mathrm{L})$$

$$\text{SII }=\text{ Platelet count }(\times 109/\mathrm{L}) \times \text{ neutrophil count }(\times 109/\mathrm{L}) /\text{ lymphocyte count }(\times 109/\mathrm{L})$$

$$\text{SIRI }=\text{ Neutrophil count }(\times 109/\mathrm{L}) \times \text{ Monocyte count }(\times 109/\mathrm{L}) /\text{ Lymphocyte count }(\times 109/\mathrm{L})$$

$$\text{AAPR }=\text{ Albumin concentration }(\mathrm{g}/\mathrm{L}) /\text{ alkaline phosphatase }(\mathrm{U}/\mathrm{L})$$

$$\text{dNLR }=\text{ Neutrophil count }(\times 109/\mathrm{L}) / (\text{white cell count }(\times 109/\mathrm{L}) -\text{ neutrophil count }(\times 109/\mathrm{L})$$

### Follow-up

Patients were evaluated every three months in the first year after RNU, every six months in the second and third years, and annually thereafter. Routine investigations included blood tests (routine blood tests and liver and renal function tests), cystoscopy, and imaging. Follow-up ranged from the date of surgery to the most recent visit or death.

### Statistical method

All the data were statistically analyzed using SPSS software (version 25.0, IBM, Chicago, IL, USA). The optimal cut-off values of SII, PNI, SIRI, and AAPR were obtained by receiver operating characteristic curves (ROC) curve and divided into high and low groups. The overall survival rate and disease-free survival rate of patients were statistically analyzed. The clinical and pathological characteristics of each group were compared and analyzed by Chi-square test. Kaplan–Meier survival analysis and the Log-rank test were used to plot and compare survival curves. Cox proportional hazard regression model was used for univariate and multivariate analysis. All tests were two-tailed, and a *P* < 0.05 was considered statistically significant.

## Results

A total of 81 patients with UTUC were included in the study, with an average age of 71.77 years (age range: 34–88). There were 43 (53.09%) males and 38 (46.91%) females. Patients with BMI ≥ 24.0 were defined as overweight, including 33 (40.74%) with BMI ≥ 24.0 and 48 (59.26%) with BMI < 24.0. There were 18 cases (22.22%) of low-grade tumors and 63 cases (77.78%) of high-grade tumors. In addition, 38 cases (46.91%) were papillary tumors, and 43 (53.09%) were invasive. There were 20 (24.7%), 16 (19.8%), 42 (51.9%), and three cases (3.7%) of T1, T2, T3, and T4 tumors, respectively. According to the ROC curve, the 81 patients with UTUC were divided into high and low groups using a PNI, SII, SIRI, and AAPR values of 48.15, 596.4, 1.45, and 0.50, respectively. In addition, three [21] and the upper limit of normal value (ULN: 250 U/L) was selected as the criteria for dNLR and LDH, respectively. The LIPI was classified as good if both indicators were normal (dNLR ≤ 3 and LDH ≤ ULN), medium if any indicator was abnormal (dNLR > 3 or LDH > ULN), and poor if both indicators were abnormal (dNLR > 3 and LDH > ULN).

**Fig. 1:**
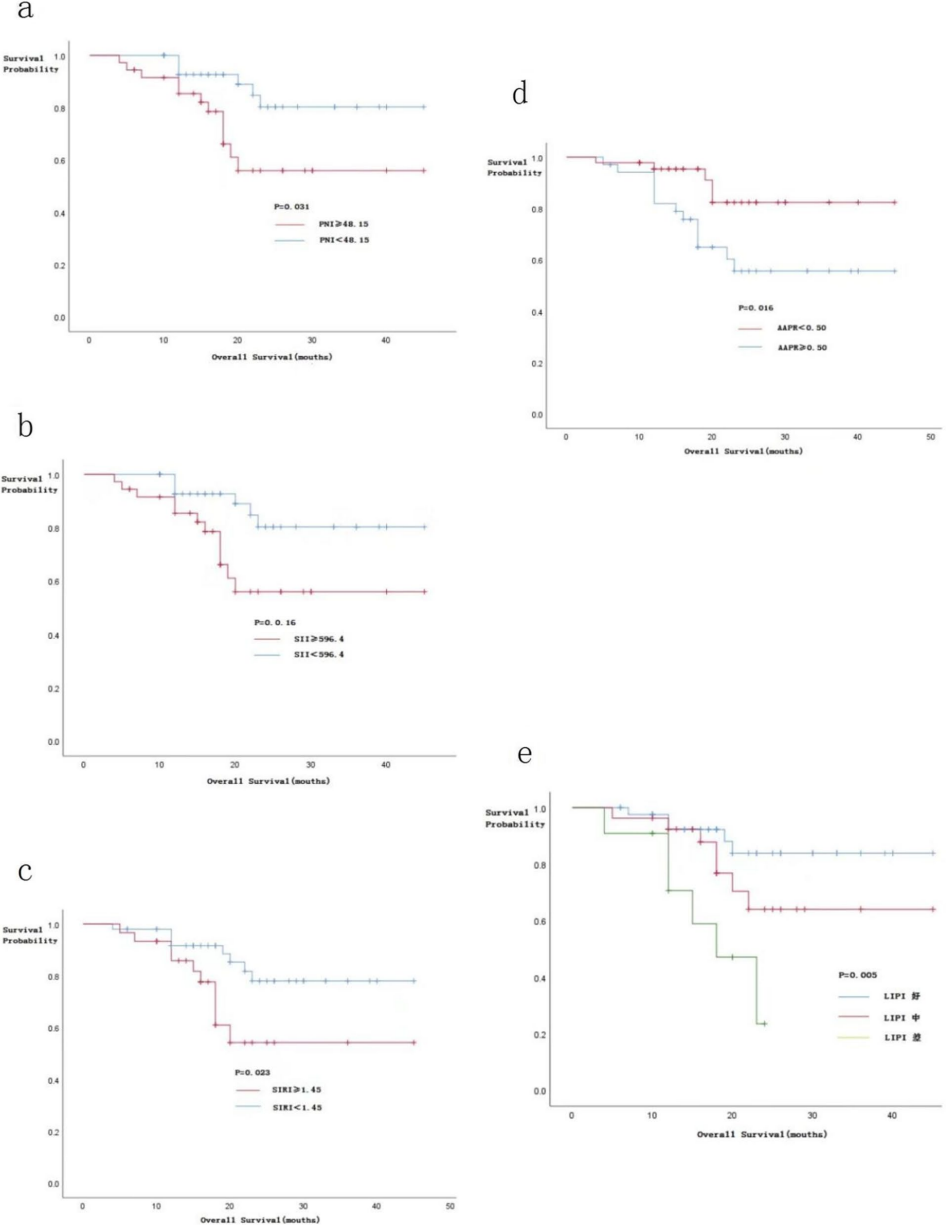
The relationship between the OS of patients with the PNI (**a**), SII (**b**), SIRI (**c**), AAPR (**d**), and LIPI (**e**)

**Fig. 2:**
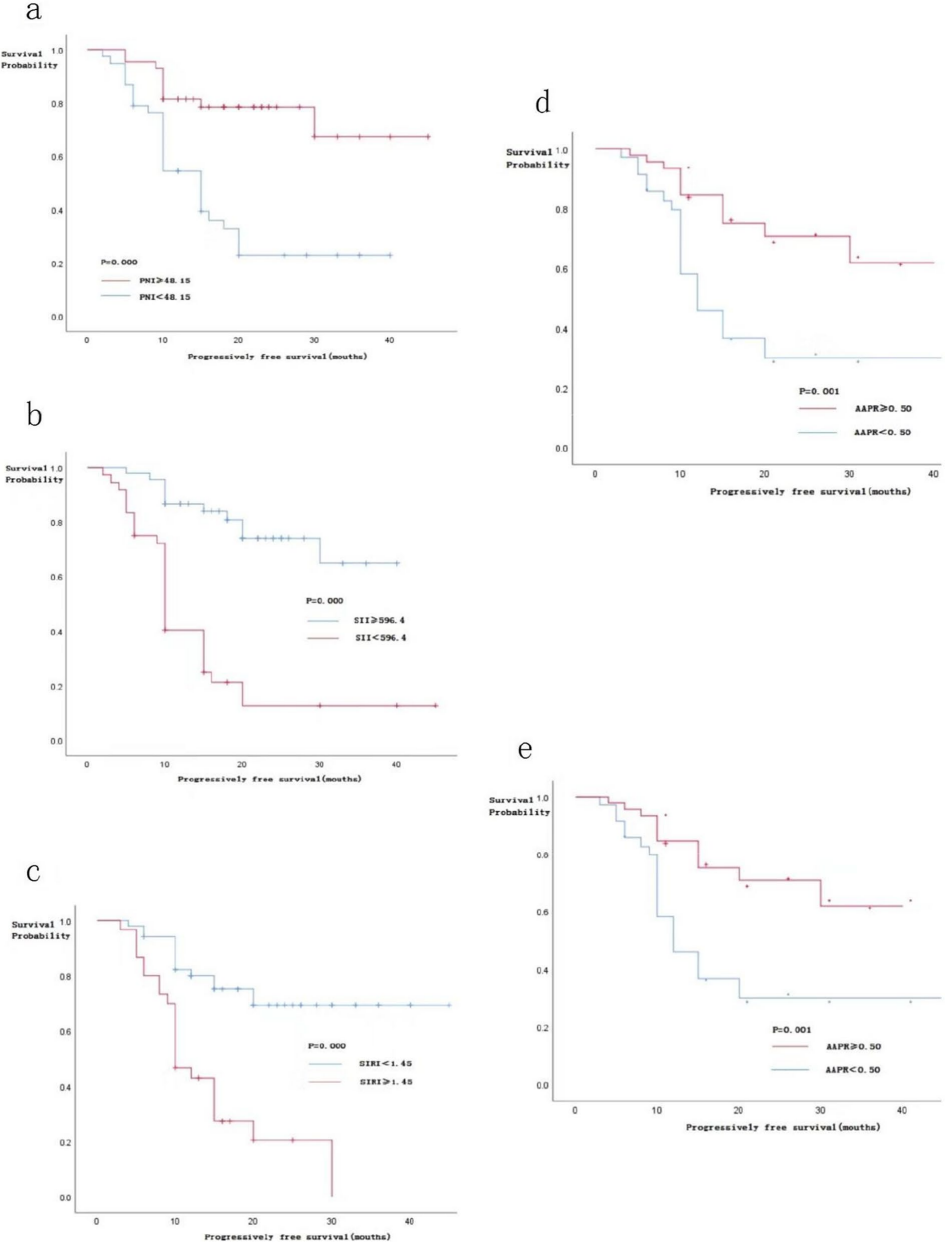
The relationship between the PFS of patients with the PNI (**a**), SII (**b**), SIRI (**c**), AAPR (**d**), and LIPI (**e**)

A total of 38 (46.91%) and 43 patients (53.09%) were divided into the low PNI (PNI < 48.15) and high PNI group (PNI ≥ 48.15), respectively. There was no statistically significant difference between PNI and age, gender, or BMI of the patients, but there was a statistically significant difference between PNI and tumor grade, classification, and stage (*P* < 0.05) (Table 1). Patients in the low PNI group had shorter OS and PFS (Figs. 1A and 2A).

**Table 1:** Relationship between the prognostic nutritional index and clinical features of upper tract urothelial carcinoma patients

		PNI		
Clinical features	Number of cases	Low	High	*P*
Sex (%)				0.157
Male	43	17 (39.5%)	26 (60.5%)	
Female	38	21 (55.3%)	17 (44.7%)	
Age (years, %)				0.365
≥ 70	49	21 (42.9%)	28 (57.1%)	
< 70	32	17 (53.1%)	15 (46.9%)	
Body mass index (BMI, %)				0.502
≥ 24.0	33	14 (42.4%)	19 (57.6%)	
< 24.0	48	24 (50.0%)	24 (50.0%)	
Pathological grading (%)				0.001
Low-grade	18	16 (88.9%)	2 (11.1%)	
High-grade	63	36 (57.1%)	27 (42.9%)	
Tumor type (%)				0.002
Papillary tumor	38	11 (28.9%)	27 (71.1%)	
Invasive tumor	43	27 (62.8%)	16 (37.2%)	
Clinical stages (%)				0.021
T1	20	5 (25.0%)	15 (75.0%)	
T2	16	5 (31.2%)	11 (68.8%)	
T3	42	26 (61.9%)	16 (38.1%)	
T4	3	2 (66.7%)	1 (33.3%)

In addition, 45 (55.56%) and 36 patients (44.44%) were assigned to the low SII group (SII < 596.4) and high SII group (SII ≥ 596.4). There was no significant statistical difference between SII and age, gender, or BMI of the patients, but there was a statistical difference with tumor grade, classification, and stage (*P* < 0.05) (Table 2). Patients in the high SII group had shorter OS and PFS (Figs. 1B and 2B).

**Table 2:** Relationship between the systemic immune-inflammation index and clinical features of upper tract urothelial carcinoma patients

		SII		
Clinical features	Number of cases	Low	High	*P*
Sex (%)				0.960
Male	43	24 (55.8%)	19 (44.2%)	
Female	38	21 (55.3%)	17 (44.7%)	
Age (years, %)				0.204
≥ 70	49	30 (61.2%)	19 (38.8%)	
< 70	32	15 (46.9%)	17 (53.1%)	
Body mass index (BMI, %)				0.544
≥ 24.0	33	17 (51.5%)	16 (49.5%)	
< 24.0	48	28 (58.3%)	20 (41.7%)	
Pathological grading (%)				0.001
Low-grade	18	16 (88.9%)	2 (11.1%)	
High-grade	63	29 (46.0%)	34 (54.0%)	
Tumor type (%)				0.028
Papillary tumor	38	26 (68.4%)	12 (32.6%)	
Invasive tumor	43	19 (44.2%)	24 (55.8%)	
Clinical stages (%)				0.007
T1	20	15 (75.0%)	5 (25.0%)	
T2	16	12 (75.0%)	4 (25.0%)	
T3	42	18 (42.9%)	24 (57.1%)	
T4	3	0 (0.0%)	3 (100.0%)	

Moreover, 51 (62.96%) and 30 patients (37.04%) were assigned to the low SIRI group (SIRI < 1.45) and high SIRI group (SIRI ≥ 1.45), respectively. There was no statistically significant difference between SIRI and the age, gender, and BMI of the patients, but there was a statistically significant difference with the tumor grade, classification, and stage (*P* < 0.05) (Table 3). Patients in the high SIRI group had shorter OS and PFS (Figs. 1C and 2C).

**Table 3:** Relationship between systemic inflammation response index and clinical features of upper tract urothelial carcinoma patients

		SIRI		
Clinical features	Number of cases	Low	High	*P*
Sex (%)				0.669
Male	43	28 (65.1%)	15 (34.9%)	
Female	38	23 (60.5%)	15 (69.5%)	
Age (years, %)				0.312
70	49	33 (67.3%)	16 (32.7%)	
< 70	32	18 (56.3%)	14 (43.7%)	
Body mass index (BMI, %)				0.567
≥ 24.0	33	22 (66.7%)	11 (33.3%)	
< 24.0	48	29 (60.4%)	19 (39.6%)	
Pathological grading (%)				0.002
Low-grade	18	1 (5.6%)	17 (94.4%)	
High-grade	63	29 (46.0%)	34 (54.0%)	
Tumor type (%)				0.000
Papillary tumor	38	6 (13.2%)	33 (86.8%)	
Invasive tumor	43	18 (41.9%)	25 (58.1%)	
Clinical stages (%)				0.000
T1	20	19 (95.0%)	1 (5.0%)	
T2	16	13 (81.3%)	3 (18.7%)	
T3	42	19 (45.2%)	23 (54.8%)	
T4	3	0 (0.0%)	3 (100.0%)	

Furthermore, 35 (43.20%) and 46 patients (56.80%) were assigned to the low AAPR group (AAPR < 0.50) and high AAPR group (AAPR ≥ 0.50), respectively. There was no statistically significant difference between AAPR and age, gender, or BMI of the patients, but there was a statistically significant difference between AAPR and tumor grade, classification, and stage (*P* < 0.05) (Table 4). Patients with low AAPR had shorter OS and PFS (Figs. 1D and 2D).

**Table 4:** Relationship between albumin-to-alkaline phosphatase ratio and clinical features of upper tract urothelial carcinoma patients

		AAPR		
Clinical features	Number of cases	Low	High	*P*
Sex (%)				0.108
Male	43	15 (34.9%)	28 (65.1%)	
Female	38	20 (52.6%)	18 (47.4%)	
Age (years, %)				0.145
≥ 70	49	18 (36.7%)	31 (63.3%)	
< 70	32	17 (53.1%)	15 (46.9%)	
Body mass index (BMI, %)				0.906
≥ 24.0	33	14 (42.4%)	17 (57.6%)	
< 24.0	48	21 (43.8%)	27 (56.2%)	
Pathological grading (%)				0.001
Low-grade	18	1 (5.6%)	17 (94.4%)	
High-grade	63	34 (54.0%)	29 (46.0%)	
Tumor type (%)				0.004
Papillary tumor	38	10 (26.4%)	28 (73.6%)	
Invasive tumor	43	25 (58.1%)	18 (41.9%)	
Clinical stages (%)				0.001
T1	20	2 (10.0%)	18 (90.0%)	
T2	16	6 (37.5%)	10 (62.5%)	

Finally, 43 (53.09%), 27 (33.33%), and 11 patients (13.58%) were in the good, medium, and poor LIPI groups, respectively. There was no statistically significant difference between LIPI and age, gender, or BMI of the patients, but there was a statistically significant difference between LIPI and tumor grade, classification, and stage (*P* < 0.05) (Table 5). Patients in the LIPI difference group had shorter OS and PFS (Figs. 1E and 2E).

**Table 5:** Relationship between lung Immune prognostic Index and clinical features of upper tract urothelial carcinoma patients

		LIPI			
Clinical features	Number of cases	Poor	Medium	Good	*p*
Sex (%)					0.233
Male	43	7 (16.3%)	17 (39.5%)	19 (44.2%)	
Female	38	4 (10.5%)	10 (26.3%)	24 (63.2%)	
Age (years, %)					0.486
≥ 70	49	5 (10.2%)	16 (32.7%)	28 (57.1%)	
< 70	32	15 (46.9%)	11 (34.4%)	6 (18.8%)	
Body mass index (BMI, %)					0.107
≥ 24.0	33	4 (12.1%)	7 (21.1%)	22 (66.7%)	
< 24.0	48	7 (14.6%)	20 (41.7%)	21 (43.8%)	
Pathological grading (%)					0.002
Low-grade	18	0 (0.0%)	2 (11.1%)	16 (88.9%)	
High-grade	63	11 (17.5%)	25 (39.7%)	27 (342.9%)	
Tumor type (%)					0.020
Papillary tumor	38	2 (5.3%)	10 (26.3%)	26 (68.4%)	
Invasive tumor	43	9 (20.9%)	17 (39.5%)	17 (39.5%)	
Clinical stages (%)					0.020
T1	20	1 (5.0%)	5 (25.0%)	14 (70.0%)	
T2	16	0 (0.0%)	8 (50.0%)	8 (50.0%)	
T3	42	8 (19.0%)	13 (31.0%)	21 (50.0%)	
T4	3	2 (66.7%)	1 (33.3%)	0 (00.0%)	

Univariate Cox proportional hazard regression showed that low PNI (hazard ratio (HR): 2.905; 95% confidence interval (CI): 1.035–8.153; *P* = 0.043), high SII (HR: 0.322; 95% CI: 0.120–0.864; *P* = 0.024), and high SIRI (HR: 0.024). Low AAPR (HR: 3.254 95% CI: 1.160–9.132, *P* = 0.025) and low LIPI groups (HR: 2.437; 95% CI: 1.330–4.466; *P* = 0.004) were effective predictors of postoperative prognosis in patients with UTUC. Multivariate Cox proportional hazard regression analysis showed that high SII (HR: 0.311; 95% CI: 0.098–0.092; *P* = 0.048) was an independent risk factor of postoperative prognosis of patients with UTUC (Table 6).

**Table 6:** Cox regression analysis of total survival time and preoperative inflammation, nutrition and immune index of upper tract urothelial carcinoma patients

Clinical index	Univariate Cox		Multivariate Cox	
	HR (95% CI)	*p*	HR (95% CI)	*p*
PNI	2.905 (1.035 ~ 8.153)	0.043	1.658 (0.500 ~ 5.497)	0.409
SII	0.322 (0.120 ~ 0.864)	0.024	0.311 (0.098 ~ 0.092)	0.048
SIRI	0.357 (0.139 ~ 0.914)	0.032	0.668 (0.219 ~ 2.044)	0.480
AAPR	3.254 (1.160 ~ 9.132)	0.025	2.672 (0.666 ~ 10.725)	0.166
LIPI	2.437 (1.330 ~ 4.466)	0.004	3.664 (0.860 ~ 15.617)	0.079

**Fig. 3:**
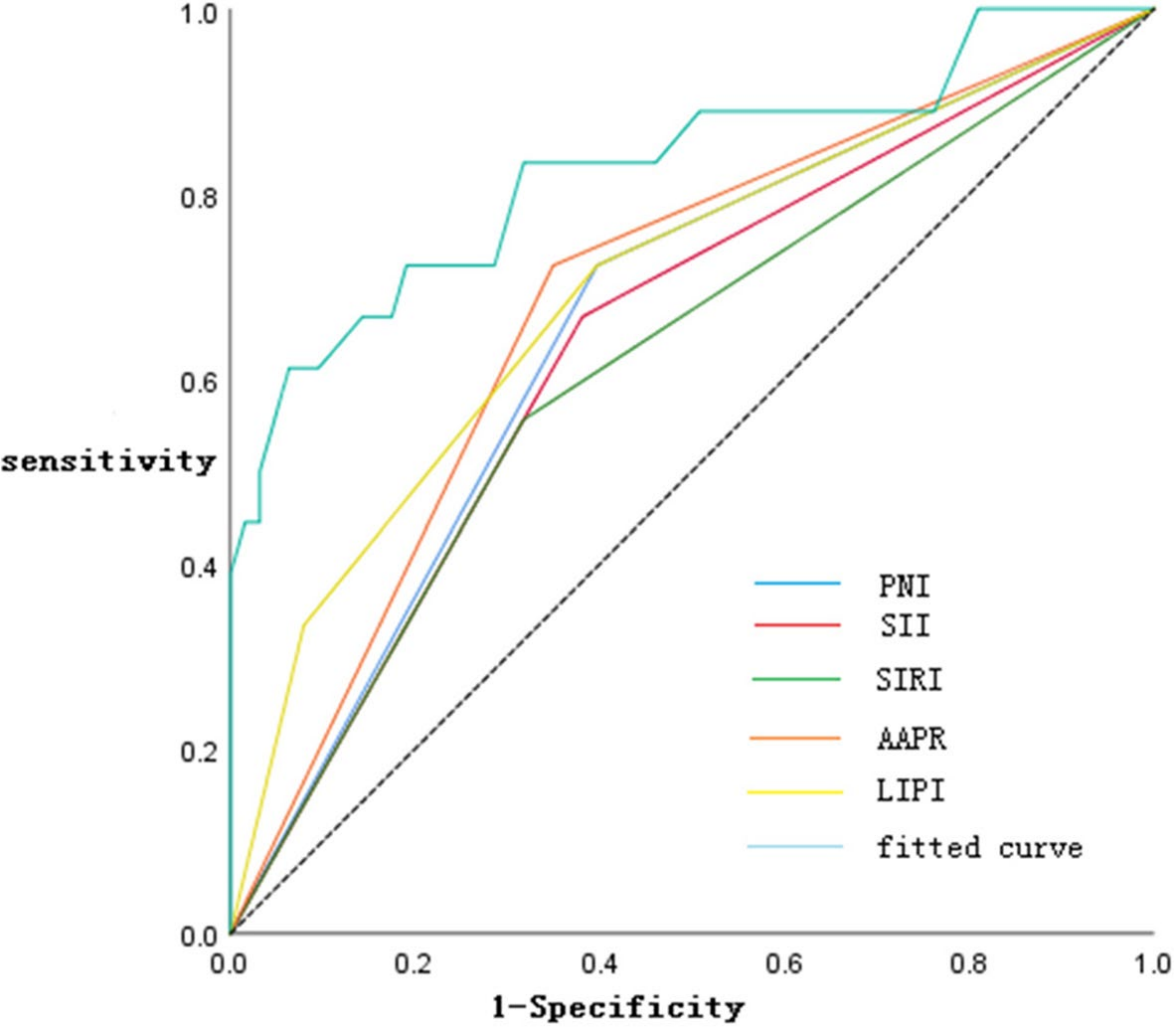
Receiver operating characteristic curve of PNI, SII, SIRI, AAPR, LIPI, and fitting index for predicting survival

This study further evaluated the prognostic value of PNI, SII, SIRI, AAPR, and LIPI fitting indexes in patients with UTUC after surgery. According to the ROC curve, the AUC of PNI, SII, SIRI, AAPR, and LIPI were 0.663, 0.643, 0.619, 0.687, and 0.700, and that of the fitting index was 0.828. Compared with PNI, SII, SIRI, AAPR, and LIPI, we found that the AUC of the fitting index was the largest, and the fitting indexes of PNI, SII, SIRI, AAPR, and LIPI could improve the prediction performance (Fig. 3).

## Discussion

UTUC, also known as upper urinary tract transitional cell carcinoma, is a rare neoplastic disease [27]. The incidence of UTUC in men is approximately twice that in women, with the highest incidence occurring in the 70–90 age group [28], broadly consistent with this study. In recent years, significant progress has been made in the diagnosis and treatment of UTUC. However, the survival and prognosis of UTUC are still poor. Some clinical indicators have also been developed to predict the prognosis of UTUC and help clinicians to select the most appropriate treatment plan for patients [29]. In this study, low PNI, high SIRI, low AAPR, and poor LIPI scores were effective predictors of PFS and OS post-UTUC, and high SII was an independent predictor of PFS and OS post-UTUC. Low PNI, high SII, high SIRI, low AAPR, and poor LIPI score may identify patients with shorter survival times. In addition, the areas under the ROC curve of PNI, SII, SIRI, AAPR, and LIPI fitting indexes were larger than those under the single prediction, indicating that the fitting indexes of PNI, SII, SIRI, AAPR, and LIPI were better than those of PNI, SII, SIRI, AAPR, and LIPI when used alone in the prediction of postoperative OS.

The mechanism of interaction between systemic inflammatory response and tumor invasion, proliferation, and metastasis is still controversial. TME includes not only cancer cells but also immune cells [30]. In 2014, Hu proposed a new inflammatory marker, SII, which integrates the three circulating immune cells, including lymphocyte, neutrophil, and platelet, and proved that it was related to the prognosis of hepatocellular carcinoma [31]. Since then, a large number of studies have found that SII can effectively predict the prognosis of various malignant tumors, such as non-small cell lung cancer, colorectal cancer, gastric cancer, esophageal squamous cell carcinoma, and pancreatic cancer [32–34].

Neutrophils, as an important type of white blood cells, are associated with congenital immune responses [35]. In the traditional view, neutrophils are mainly responsible for the host's defense function, immune regulation function, and tissue damage, while their role in tumor progression is ignored [36]. Studies have shown that in malignant tumors, neutrophils will stay in the TME for a longer time in the presence of pro-inflammatory substances, thus playing a significant role in promoting the tumor [37].

In 1865, Trousseau pioneered research on the correlation between platelets and cancer. Since then, more studies have shown that platelet counts of cancer patients may be increased, especially in advanced and advanced patients [38]. Platelets play a role in angiogenesis and cancer cell development [39]. Studies have found that more than 30% of cancer patients have platelet abnormalities, which affect the prognosis to some extent [40].

The anti-tumor effect of lymphocytes plays a key role in the progression of malignant tumors [41]. After recognizing the tumor antigen, the antigen-presenting cells at the tumor site activate T lymphocytes to reach the tumor site through the blood circulation and attack cancer cells and release the tumor antigen to kill the cancer cells by increasing the tumor immune-circulation capacity [42]. Circulating lymphocytes have an anti-tumor effect and promote the death of cytotoxic cells by producing cytokines, such as INF-γ and TNF-α [43]. In addition, a previous study reported that high SII was associated with increased levels of inflammatory cytokines and chemokines in serum [44]. Therefore, elevated SII levels suggest a marked inflammatory response and a low immune response, and patients with elevated SII levels have a relatively poor postoperative prognosis. This is also consistent with the results of this study.

Cancer is often accompanied by symptoms of malnutrition, which greatly affect the prognosis of patients. Malnutrition runs through the whole process of cancer and has a serious negative impact on the prognosis of patients. PNI, combined with serum protein level and lymphocyte count, has been used to assess the nutritional status of patients and proved to be an independent risk factor for the prognosis of various cancers, including colon cancer [45]. This study showed that PNI could be used as a risk factor, and the survival time of the high PNI group was longer than that of the low PNI group, which was basically consistent with the results in the previous study.

SIRI, developed by Qi, is a simple and practical inflammatory index to predict the prognosis of patients with advanced pancreatic cancer and can be used to monitor the local immune response and systemic inflammatory state of tumor patients [8]. Cytokines and chemokines secreted by inflammatory cells during a systemic inflammatory response, such as IL-6, TNF-α, and bone marrow growth factor, can enhance the invasion, proliferation, and metastasis of cancer cells and further lead to immune escape and tumor cells' tolerance to chemotherapy drugs. Many studies have applied SIRI to the prognostic evaluation of other cancer patients and showed that SIRI is related to the clinicopathological features and prognosis of various tumors [9, 10, 46]. This study showed that SIRI can be used as a risk factor, and the survival time of the low SIRI group is longer than that of the high SIRI group.

AAPR is a simple and effective integration of serum albumin and alkaline phosphatase (ALP), two serum biomarkers, to form a new and effective prognostic indicator. A systematic evaluation of 29 epidemiological studies by Digant showed that serum albumin level before treatment was an independent predictor of survival of tumor patients and had important significance for evaluating the prognosis of tumor patients [47]. It has been found that ALP is an indicator of advanced stages of cancer, and the level of ALP in cancer patients increases with bone metastasis [48]. Many studies have explored the predictive value of AAPR in tumors, and its clinical significance has been verified in a variety of malignant tumors, which showed that low preoperative AAPR is an independent indicator of poor prognosis for patients after cancer surgery [20, 49].

dNLR is a new indicator found in recent studies, and it simultaneously contains the most representative inflammatory immune cells, such as leukocytes, neutrophils, lymphocytes, and monocytes. It has been reported that it is related to the prognosis of various tumors, such as urinary tumors [50] and gastrointestinal tumors [51]. LDH is a key enzyme for the conversion of pyruvic acid into lactic acid during glycolysis and is also an enzyme necessary for maintaining anaerobic glycolysis in tumors [52]. LIPI score, as the combined indicator of LDH and dNLR, was first proposed by Mezquita in 2018 [21]. A large number of studies have proved that the LIPI score was related to PFS and OS, and a poor LIPI score was a poor prognostic factor for patients [53, 54], consistent with the results of this study.

Our study has several shortcomings. First, there was selection bias due to the small sample size and low incidence of UTUC in a single-center retrospective study. Second, although we excluded patients with known liver, kidney, and bone disorders to limit confounding factors, patients with undetected liver, kidney, or bone disorders may be mistakenly enrolled in the study. Third, follow-up time was limited. Fourth, according to the current research, there is no unified method to determine the optimal cut-off point of preoperative peripheral blood index. The critical values determined in different studies vary, resulting in a biased conclusion of the optimal cut-off value. Future large-sample prospective multicenter clinical studies are needed to evaluate the prognostic values of SII, SIRI, PNI, AAPR, and LIPI.

Interestingly, there are also a number of nutritional indicator parameters that can be used to assess the nutritional status of oncology patients. In addition to the common body mass index (BMI), Magnano M. et al. [55]used the Buzby Nutritional Risk Index (NRI) for nutritional assessment of head and neck cancer patients, and the NRI proved to be a useful tool.The BMI assessment of nutritional status only identifies malnourished patients, but it excludes all overweight and obese head and neck cancer patients. In contrast, the NRI correctly identifies malnourished and overweight/obese patients as ‘malnourished’ subjects. Better identification and tracking of specific dietary measures for all malnourished patients. Nutritional status assessment of UTUC patients can be further optimised.

## Conclusions

PNI, SIRI, AAPR, and LIPI are potential postoperative prognostic predictors for patients with UTUC and can be used for the prognostic evaluation of patients after UTUC surgery. SII is an independent risk factor for postoperative survival in patients with UTUC. The fitting indexes of SII, PNI, SIRI, AAPR, and LIPI were more predictive for postoperative patients with UTUC than individual indexes.

## Data Availability

The raw data supporting the conclusions of this article will be made available by the authors, without undue reservation. Please contact the author Yong Ou, email: 675893648@qq.com.
