# Correction: Analysis of preoperative nutrition, immunity and inflammation correlation index on the prognosis of upper tract urothelial carcinoma surgical patients: a retrospective single center study

**DOI:** 10.1186/s12893-026-03911-2

**Published:** 2026-06-18

**Authors:** Yong Ou, Yang Zheng, Dong Wang, Shangqing Ren, Yisha Liu

**Affiliations:** 1 Department of Robotic Minimally Invasive Surgery Center, ichuan Academy of Medical Sciences and Sichuan Provincial People’s Hospital and Affiliated Hospital of the University of Electronic Science and Technology of China, Chengdu, 610072 China; 2 Department of Urology, Xichang People’s Hospital, Xichang, Sichuan China; 3 Department of Pathology, Sichuan Academy of Medical Sciences and Sichuan Provincial People’s Hospital, Chengdu, China

**Correction: BMC Surg 24, 208 (2024)**

**https://doi.org/10.1186/s12893-024-02496-y**

In article [1], The authors have corrected the following errors in their article “Analysis of preoperative nutrition, immunity and inflammation correlation index on the prognosis of upper tract urothelial carcinoma surgical patients: a retrospective single center study”, published in BMC Surgery on July 16, 2024: Page 3, paragraph 3, line 5: “The overall survival rate and disease-free survival rate of patients were statistically analyzed.” was added after the sentence “The optimal cut-off values of SII, PNI, SIRI, and AAPR were obtained by receiver operating characteristic curves (ROC) curve and divided into high and low groups.”

The original article has been corrected.
